# Cardiovascular responses to heat and cold exposure are altered by preterm birth in guinea pigs

**DOI:** 10.14814/phy2.70098

**Published:** 2024-10-22

**Authors:** Ryan Phillip Sixtus, Clint Gray, Heather Barnes, Emily Sarah Jane Paterson, Mary Judith Berry, Rebecca Maree Dyson

**Affiliations:** ^1^ Department of Paediatrics and Child Health University of Otago Wellington New Zealand; ^2^ Department of Surgery and Anaesthesia University of Otago Wellington New Zealand; ^3^ Present address: Department of Biological and Life Sciences Cardiff University Wales UK

**Keywords:** cardiovascular, cold, heat, preterm birth

## Abstract

Adversity early in life can modify the trajectory for disease risk extending decades beyond the event. Preterm birth produces persistent cardiovascular alterations that may appear maladaptive in adulthood. We have previously hypothesized that those born preterm may exhibit cardiovascular vulnerability in the climate change context. Further, this vulnerability may be present as early as childhood. We aimed to identify the early signs of cardiovascular dysfunction at childhood‐equivalent age using our animal model of preterm birth. Using a whole‐body thermal stress test, guinea pigs aged 35‐d and 38‐d (equivalent to 8–10‐year‐old children) and born at term or preterm gestations were exposed to progressive hyper‐ (*T*_C_ = 41.5°C) and hypo‐thermia (*T*_C_ = 34°C; normothermia *T*_C_ = 39°C). Comprehensive cardiovascular monitoring included ECG, blood pressure, microvascular perfusion, blood gas, and catecholamine profile, as well as skin and core body temperature. Preterm‐born animals exhibited attenuated vascular responses to hyperthermic stress, and a significant elevation in systolic blood pressure in response to hypothermic stress. Such responses are similar to those observed in elderly populations and indicate the presence of cardiovascular dysfunction. This is the first study to demonstrate the impact of preterm birth on the cardiovascular response to both heat and cold stress. Further, this dysfunction has been observed at an earlier age than that achievable using traditional stress testing techniques. The present findings warrant further investigation.

## 1 INTRODUCTION

Annually, fifteen million infants are born preterm globally (Lewandowski et al., 2020). That is, they are born before 37 weeks completed gestation. Due to persistent structural and function changes incurred as a result of their early birth (e.g., altered cardiac geometry and size, reduced and stiffened arteries and altered microvascular density (Schuermans & Lewandowski, 2022; Sixtus et al., 2024)), those born preterm are considered to possess an independent non‐modifiable risk factor for cardiovascular disease (CVD) (Crump, 2020). These cardiovascular changes following preterm birth have been linked with hypertension, ischemic heart disease, and heart failure in adulthood (Bavineni et al., 2019; Crump, 2020; Lewandowski et al., 2020). While the mechanisms are not fully elucidated, candidate pathways include cardiac and vascular structural changes, systemic inflammation, and autonomic imbalance (Bavineni et al., 2019); factors considered causative in the etiology of cardiovascular dysfunction in elderly and CVD populations (Libby et al., 2016).

While the link between prematurity and cardiovascular dysfunction and disease risk has been established, the functional capacity of the preterm cardiovascular system is less well known. Those few studies examining the cardiovascular stress response have demonstrated reduced exercise capacity in childhood (Smith et al., 2008; Welsh et al., 2010), and dysfunctional exercise responses as early as adolescence (Haraldsdottir et al., 2019; Huckstep et al., 2018; Macdonald et al., 2021; Williamson et al., 2022). The dysfunctional exercise stress responses include impaired ejection fraction (Huckstep et al., 2018), reduced stroke volume (Macdonald et al., 2021), elevated pulse wave velocity—a marker of arterial stiffness—and blood pressure (Barnard et al., 2020). There remain many unknowns in this population, not the least of which are the sex‐ or gestation‐specific responses. In the only study to examine the response to stressors other than exercise—namely hypoxia—preterm‐born adults exhibited blunted microvascular responses, and elevated oxidative stress (Manferdelli et al., 2023). Given that climate change is driving intensification of thermal extremes, it is important examine if these dysfunctional preterm traits may contribute to vulnerability in the climate change context (Sixtus, Gray, et al., 2023).

Previous studies investigating the response to temperature in children and adults born preterm have focused on thermally induced pain and temperature perception (Iversen et al., 2018; Vederhus et al., 2012; Walker et al., 2009). While such studies indicate a possible thermosensory impairment persisting beyond the neonatal period (Vederhus et al., 2012; Walker et al., 2009), the actual cardiovascular “stress” (e.g., heart rate, blood pressure) remains unknown. In other populations with known cardiovascular vulnerability, such as the elderly, impaired thermosensitivity is associated with lability of core body temperature (*T*_C_) and increased risk of thermal injury (Holowatz et al., 2010), particularly in response to prolonged thermal stress, or extreme weather events (heat waves, cold snaps) (Desai et al., 2023).

We have previously hypothesized that the combination of poor thermal sensitivity and altered cardiovascular stress responses places those born preterm at an elevated risk during thermal extremes (Sixtus & Bailey, 2023; Sixtus, Gray, et al., 2023). Because of the complexities parsing out specific effects of prematurity from other factors (diet, heredity, clinical procedures), animal models allow a “pure” examination of the long‐term effects of preterm birth (for more, see review by Morrison et al. (2018)). We therefore aimed to examine the cardiovascular response to heat and cold using our established guinea pig model of preterm birth (Berry et al., 2015) and our validated thermal stress tests (Sixtus, Berry, et al., 2023). We hypothesized that preterm‐born guinea pigs would exhibit impaired vasomotor reactivity and elevated cardiovascular stress when exposed to progressive hyper‐ or hypo‐thermia.

## 2 METHODS

### 2.1 Ethical approval

All procedures were approved by the University of Otago, Wellington Animal Ethics Committee (Ethics Approval number: AUP 18‐214) and conformed to Health Research Council of New Zealand code of practice for the care and use of animals for scientific purposes. The study is reported according to the ARRIVE guidelines (Kilkenny et al., 2010).

### 2.2 Animals

Thirty‐one age‐ and sex‐matched juvenile Dunkin Hartley guinea pigs were included in this study (term‐born (*n* = 16), preterm‐born (*n* = 15); Table 1). Term‐born animals were selected from time‐mated litters and delivered between 67 and 70 d gestation. Preterm pups were delivered at 62 d gestation from time‐mated pregnant dams, using our previously described protocol (Berry et al., 2015). Equivalency of this gestation to 29–30 weeks' gestation in humans has been previously established (Dyson et al., 2012, 2014). Weaned animals (>21d) were housed in sex‐specific pens, in a 12:12 h. light‐ and temperature‐controlled environment (~17°C–20°C), with ad libitum access to standard guinea pig chow (Specialty Feeds, Glen Forrest, Australia), and vitamin C‐enriched water.

**TABLE 1:** Baseline animal characteristics.

	Term		Preterm		*p*
	Male	Female	Male	Female	
Birth weight (g)	98.5 ± 7.9	96.6 ± 10.5	73.0 ± 7.9	73.4 ± 7.8	††††
PI (kg m^3^)	10.9 ± 0.6	10.8 ± 1.3	12.2 ± 0.7	11.8 ± 1.4	††††
Weight and PI at first challenge (CPNA35)					
Weight (g)	357.7 ± 40.5	343.7 ± 21.2	359.5 ± 28.5	307.1 ± 47.5 *	
PI (kg m^3^)	12.6 ± 1.1	12.5 ± 1.4	13.0 ± 2.1	12.1 ± 1.3	
Heating challenge					
T_re_ (°C)	38.9 ± 0.6	39.2 ± 0.4	38.9 ± 0.3	39.1 ± 0.4	
HR (b min^−1^)	378 ± 10	367 ± 15	382 ± 12	370 ± 16	*
RR (breaths min^−1^)	86 ± 16	77 ± 10	82 ± 35	90 ± 12	†
SBP (mmHg)	58 ± 4	57 ± 7	63 ± 8	62 ± 6	†
Dist. perfusion (PU)	246 ± 147	292 ± 224	187 ± 106	236 ± 152	
Prox. perfusion (PU)	219 ± 129	156 ± 74	116 ± 38	138 ± 65	
Cooling challenge					
T_re_ (°C)	39.0 ± 0.4	39.1 ± 0.5	38.9 ± 0.3	39.0 ± 0.5	
HR (b min^−1^)	386 ± 14	369 ± 19	375 ± 8	367 ± 16	*
RR (breaths min^−1^)	80 ± 11	79 ± 21	83 ± 10	80 ± 12	
SBP (mmHg)	57 ± 5	57 ± 4	60 ± 10	62 ± 10	
Dist. perfusion (PU)	244 ± 141	322 ± 132	283 ± 169	203 ± 86	
Prox. perfusion (PU)	132 ± 120	134 ± 60	123 ± 38	132 ± 49	

*Note*: Data are presented as mean ± SD. All groups are *n* = 8, except preterm females (*n* = 7). Ponderal index (PI) = Weight (kg)/Length cubed (m^3^; crown‐rump + hind limb + hock toe). Weight and PI did not differ between challenge dates and are therefore only presented for first challenge. Statistics performed using 2‐way ANOVA (gestation×sex). Gestation effect denoted by †(*p* = 0.05–0.02) and tttt (*p* < 0.0001), sex effect denoted by *.

### 2.3 Instrumentation and equipment

Thermal challenges were performed using a custom‐made water perfused wrap connected to a precision‐controlled water bath (R‐1, ANOVA, Texas, USA), circulating water at 5 L min^−1^, as described previously (Sixtus, Berry, et al., 2023). Cardiovascular assessments consisted of heart rate (HR), systolic blood pressure (SBP), and proximal (interscapular) and distal (ear) microvascular perfusion, assessed using three‐lead ECG (needle electrodes, ADInstruments, Dunedin, New Zealand), non‐invasive blood pressure (ADInstruments), and laser Doppler flowmetry (Probe 457, Periflux 5001, Primed, Jarfalla, Sweden), respectively. Respiration was assessed using a pulse transducer (ADInstruments) positioned underneath the prone animal. Core (T_C_) temperature was measured using a rectal thermistor (RET‐1; ADInstruments) inserted 6 cm past the anus and four skin thermistors (*T*_sk_) were applied to the left ear, back, rump, and hind foot acral skin. Conscious baseline measures excluded skin thermistors but included all other parameters. Blood collection for blood glucose concentration (CareSensN Glucometer, Pharmaco Ltd., Auckland, New Zealand), blood gas analysis (CG4+, iStat, Abbott Point of Care, Princeton, USA) and circulating catecholamine analysis (3‐CAT Research ELISA, BA E‐5600R, LDN, Germany) was performed following conscious measures of cardiovascular function, immediately before anesthetizing animals. Blood was taken from the right ear to avoid damage to the microvascular perfusion assessment site. Arterialised capillary blood from the ear has been shown to provide an adequate representation of arterial blood gas parameters (Zavorsky et al., 2007).

### 2.4 Protocol

Thermal challenges were completed in a randomized order on corrected postnatal ages (postnatal age expressed relative to term “due date”; CPNA)‐35 and CPNA‐38 with a three‐day recovery between challenges (e.g., CPNA35: heating challenge; CPNA38: cooling challenge). Prior to each challenge, guinea pigs were fasted (≈4 h). Conscious baseline cardiovascular assessments were conducted under 5 mg kg^−1^ alfaxalone sedation (10 mg ml^−1^, Alfaxan Multidose, Jurox, UK (Sixtus, Pacharinsak, et al., 2021)), followed by blood collection, prior to each challenge.

The thermal challenge has been described previously (Sixtus, Berry, et al., 2023). In brief, animals were lightly anesthetized using an isoflurane—70% nitrous oxide mix (Sixtus, Gray, et al., 2021) and secured within the water‐perfused wrap. Following titration of anesthetic and stabilization of core (*T*_C_) and skin (*T*_sk_) temperature (*T*_C_ ≈38.0°C; *T*_sk_ = 36.00 ± 0.25°C), the thermal challenge was initiated. The heating challenge consisted of a 1.0°C min^−1^ bath temperature (*T*_bath_) ramp to a maximum of 44.0°C, maintained until *T*_C_ achieved 41.5°C, whereupon recovery cooling was initiated. During the cooling challenge, *T*_bath_ was reduced 1°C min^−1^ to 15.0°C, maintained until *T*_C_ achieved 34.0°C, recovery rewarming was then performed. Target *T*_C_ thresholds for both challenges were determined during pilot testing to produce pronounced hyper‐ and hypo‐thermia without excessive physiological compromise (Sixtus, Berry, et al., 2023). Recovery *T*_bath_ was set to 35°C in both challenges and maintained for 30 min before anesthesia was withdrawn and blood collected. Post‐challenge blood samples were collected for blood gas (CG4+ cartridge, Abbott Point‐of‐Care) and catecholamine analyses (3‐CAT Research ELISA) at 30 min (once roused from anesthesia), 3 h and 24 h from completion of the study. Animals were monitored in a quilt lined cage until the 3‐h blood collection, after which they were returned to their home cage. Daily welfare monitoring (weight, behavior, food intake, and fluid balance) was performed post‐challenge to ensure full recovery prior to initiating the second thermal challenge.

### 2.5 Data analysis

#### 2.5.1 Data reduction

Physiological measures were sampled continuously throughout anesthesia. ECG, SBP, respiration, *T*_C_ and *T*_sk_ were sampled using PowerLab (ADInstruments, Dunedin, NZ) at 1 k s^−1^, with an analogue notch filter applied, and recorded in LabChart (ADInstruments). HR and respiration were derived using peak‐to‐peak analysis. Rate pressure product (RPP), a surrogate for myocardial oxygen demand (Ikaheimo, 2018), was calculated using the equation RPP=HR×SBP, described by Romanovsky and Blatteis (Romanovsky & Blatteis, 1996).

Proximal and distal *T*_sk_ were generated from averaged interscapular and rump *T*_sk_, and foot and ear *T*_sk_, respectively. Skin thermistors were calibrated prior to each session. Laser Doppler flowmetry (LDF) collected alongside central cardiovascular assessments was measured at 32 Hz with a time constant of 0.03 s, assessed in arbitrary perfusion units (PU). LDF measures were calibrated prior to each session (PF1000 calibration device, Perimed). Collected LDF data was then logged for analysis. Representative, artifact‐free data was assessed in 1 min blocks every 5 min across all continuous physiological measures. Dehydration from insensible heat loss was calculated from weight measured immediately pre‐ and post‐challenge, using the following equation:

$$\text{Percentage dehydration}=\frac{\left(\mathrm{Pre}\;\text{heat}\;\mathrm{BW}-\text{Post heat}\;\mathrm{BW}\right)}{\begin{array}{c} \mathrm{Pre}\;\text{heat}\;\mathrm{BW}\;\\ \; \end{array}}\times 100$$

#### 2.5.2 Statistical analyses

**FIGURE 1:**
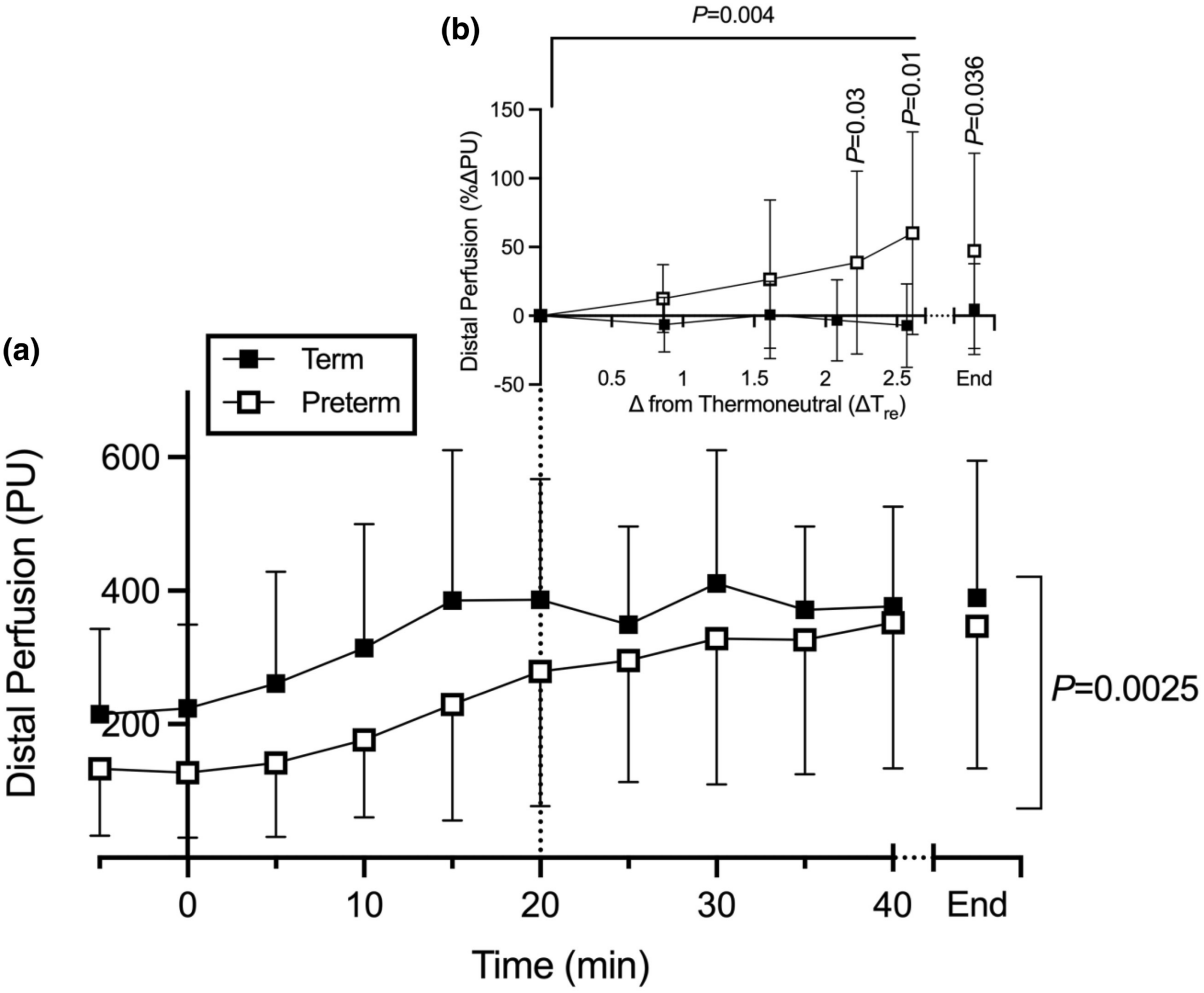
Distal (ear) perfusion response (a) across the duration of the heating challenge, and (b) as percentage change from thermoneutral (39°C, 20 min) to challenge end. Data are presented as mean ± SD. Repeated measures analysis performed on log‐transformed perfusion data using Bonferroni‐corrected mixed effects analysis. No sex effect observed by 3‐way (time × gestation × sex) mixed effects (*p* = 0.7884), therefore sex‐specific results consolidated in two‐way mixed effects (time × gestation). Challenge duration differed among animals (due to starting *T*_C_ and max *T*_C_ threshold of 41.5°C); group numbers are as follows (Term‐Preterm animals, respectively): 0–25 min: 15–15, 30 min: 14–15, 35 min: 13–15, 40 min: 5–11. “End” denotes the final timepoint for each animal; statistics performed on consolidated “End” timepoint using two‐way ANOVA (gestation × sex).

**FIGURE 2:**
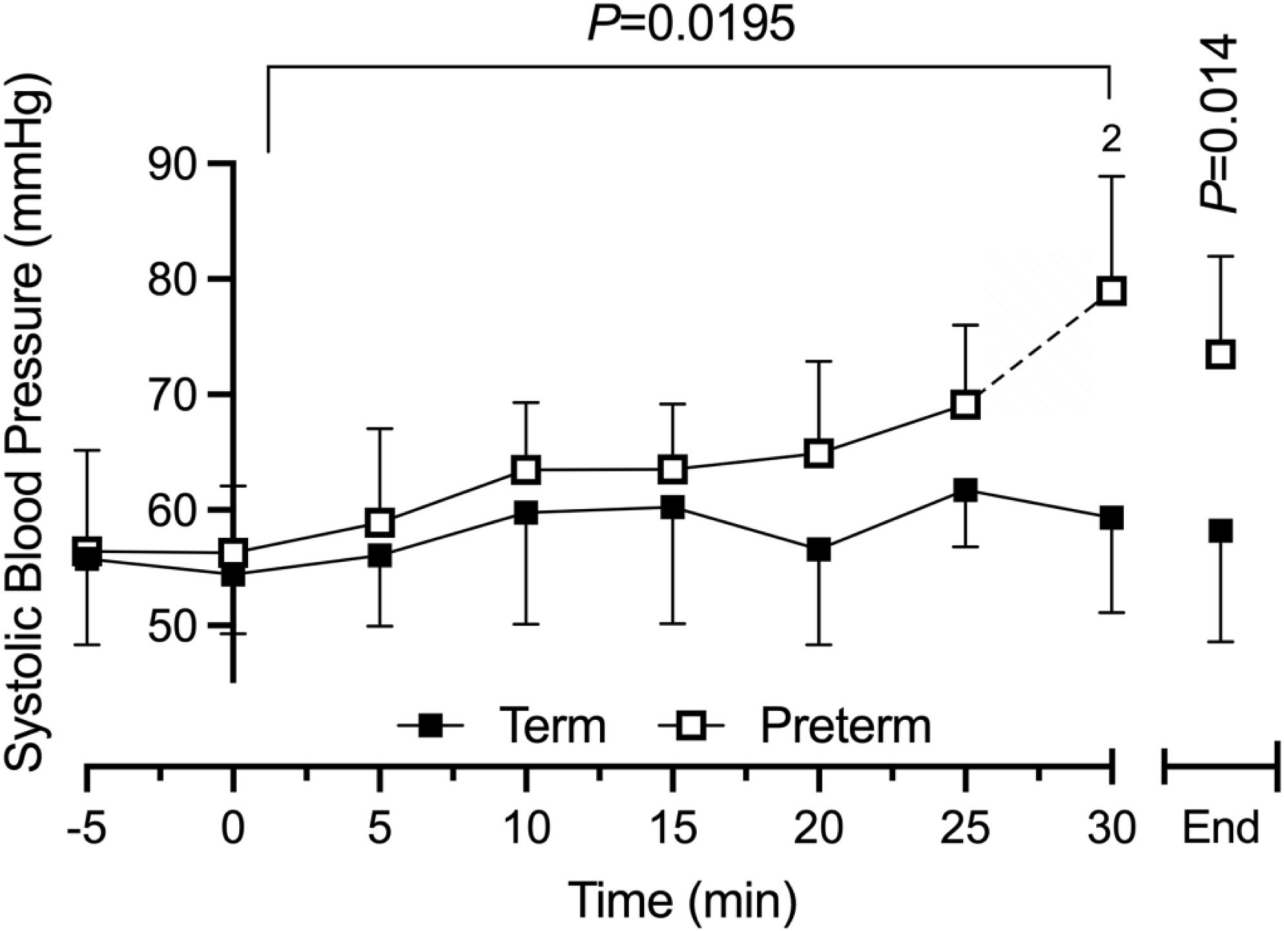
Systolic blood pressure (mean ± SD) response to progressive hypothermia in term and preterm‐born animals (CPNA35). “End” denotes the consolidated final challenge timepoint for each animal (*T*_re_ = 34°C). *p* values represent time × gestation effect and gestation effect in “End” timepoint. Analyses by two‐way ANOVA with time × gestation for repeated measures and sex × gestation for “End.” Due to signal drop‐out from shivering thermogenesis, blood pressure was only obtained for “2” of 5 preterm animals at 30 min (term *n* = 5). “*N*” numbers across challenge duration are as follows (Term‐Preterm animals, respectively): −5 min: 12–11, 0 min: 15–12, 5 min: 15–12, 10 min: 16–10, 15 min: 15–11, 20 min: 8–10, 25 min: 6–6, 30 min: 5–2, “End”: 9–7.

Data was analyzed using two‐way ANOVA for characteristics (gestation×sex; Table 1) and three‐way mixed effects analysis for repeated measures (gestation×sex×time; Figures 1 and 2) in GraphPad Prism (GraphPad Software, CA, USA). To account for differences in the cessation temperature (e.g., 41.5°C or 34°C), a discrete timepoint, “End,” was created to assess maximal physiological stress. This timepoint was analyzed by two‐way ANOVA. Data are presented as mean ± SD, and significance set at *p* < 0.05.

## 3 RESULTS

### 3.1 Animal characteristics

Animal characteristics are presented in Table 1 alongside baseline (waking) cardiovascular characteristics. Preterm animals were significantly smaller at birth (weight and PI *p* < 0.0001, respectively), but were of comparable weight and size at time of first challenge (weight: *p* = 0.1829 and PI: *p* = 0.984, respectively). Within preterm animals, female animals exhibited less catch‐up weight gain by the time of first challenge (CPNA35) than their male counterparts (difference, weight: 52.3 g, *p* = 0.0384). Baseline physiological parameters were similarly comparable (Table 1).

### 3.2 Heating challenge—Attenuated vascular response

Distal microvasculature displayed a significant time and gestation interaction (time xGA: *p* = 0.0025; Figure 1a). No sex effect was observed (*p* = 0.7884). Preterm animals began the challenge with lower distal perfusion (81 PU) despite comparable thermal profiles (grouped *T*_sk_: 35.8 ± 0.4°C, grouped *T*_C_: 37.0 ± 0.3°C; Figure S1). T_C_ increased rapidly across the challenge, achieving waking T_C_ (group *T*_C_: 39.0 ± 0.4°C) by 20 min (Figure 1: dotted line). While term‐born animals exhibited an immediate and rapid increase in distal perfusion, achieving maximum by 15 min (0–15 min slope: 11 PU min^−1^), those born preterm exhibited an attenuated response (0–15 min slope: 7 PU min^−1^), and never achieved a maximum plateau (20–40 min slope, preterm: 4 PU min^−1^, term: 0 PU min^−1^). Further demonstrating this attenuated response, when expressed relative to thermoneutral (~39°C), term animals exhibited no further increases in perfusion (challenge end/41.5°C: −0.2%), whereas preterm animals exhibited a significant 60% increase in perfusion between 39°C and 41.5°C (Figure 1b, gestation effect *p* = 0.004; time effect: *p* < 0.0001). Although there was no significant difference in other physiological or hematological parameters between animals born preterm or at term, (Figure S2; Table S1), those born preterm appeared to dehydrate to a greater extent based on body weight change pre‐ and post‐challenge (term weight change: −2.5 ± 2.1%; preterm weight change: −4.0 ± 2.5%).

### 3.3 Cooling challenge—Excess blood pressure

Systolic blood pressure (SBP) exhibited a significant time and gestation interaction across the duration of the challenge (time × GA: *p* = 0.0195; Figure 2). No sex effect was observed (*p* = 0.146). Baseline SBP of animals born at term and preterm was 55.8 ± 7.5 mmHg and 56.4 ± 8.7 mmHg, respectively. Whereas term‐born animals maintained a similar SBP throughout challenge (“End”: 58.2 ± 9.6 mmHg; *p* = 0.9437), SBP of preterm‐born animals rose significantly (25 min: 69.2 ± 6.9 mmHg, *p* = 0.0256; “End”: 73.5 ± 8.5 mmHg, *p* = 0.038). When aligned for final challenge measurement (Figure 2: “End”), SBP of preterm animals was significantly above that of term animals (*p* = 0.014). The increase in SBP, in the absence of increasing HR, produced a reciprocal increase in RPP of preterm‐born animals though this did not reach statistical significance (time effect: *p* = 0.069; GA: *p* = 0.052; time × GA: *p* = 0.076). Additional physiological parameters are presented in Figures S3 and S4.

## 4 DISCUSSION

We have demonstrated for the first time that the cardiovascular response to both heat and cold stress is altered following preterm birth. Healthy juvenile guinea pigs born preterm exhibited an attenuated rise in microvascular perfusion during progressive hyperthermic stress, and an excess rise in SBP during progressive hypothermic stress. This amplified SBP response to cold stress supports our hypothesis that structural abnormalities are the dominant driver of cardiovascular dysfunction at this age (Sixtus et al., 2024; Sixtus, Gray, et al., 2023). To date, studies investigating the cardiovascular stress response in childhood are sparse and have not demonstrated the same gestational age‐dependent response (Smith et al., 2008; Welsh et al., 2010). However, within any clinical population, especially during childhood, the stressor utilized to assess hemodynamic response are necessarily far less potent than the hyper‐ and hypo‐thermic challenges described presently.

### 4.1 Attenuated vasomotor response

In the present study preterm‐born animals exhibited an attenuated vascular response to hyperthermic stress and never achieved a maximum plateau in distal perfusion. The vascular response is the first thermoeffector response when exposed to heat, aimed at mitigating a rise in *T*_C_ (Schlader et al., 2018), and therefore should respond at the first detectable signs of rising temperature. The primary “effector” of this response is the arteriovenous anastomoses located in acral—non‐hairy—skin sites, such as the ears, hands, and feet (Taylor et al., 2014). It is only following the vasomotor responses' failure to mitigate these changes in T_C_ that further thermoeffectors (e.g., sweating) are recruited to restore normothermia despite changes in ambient temperature (Schlader et al., 2018).

Attenuated vascular responses have been observed in elderly and those with CVD (Gravel et al., 2021; Kenny et al., 2010). The mechanisms may differ slightly, but include decreased thermosensitivity (Guergova & Dufour, 2011), endothelial dysfunction (Holowatz & Kenney, 2007, 2010), autonomic dysregulation (Buford, 2016), and microvascular rarefaction (Gravel et al., 2021). These impairments increase the physiological stress experienced in response to heat exposure within these populations and are ultimately responsible for their elevated cardiovascular risk during high temperatures (Desai et al., 2023; Gravel et al., 2021; Holowatz et al., 2010). Within the preterm population, thermosensitivity appears to be reduced in childhood (Hermann et al., 2006; Walker et al., 2009), but perhaps not beyond (Iversen et al., 2018; Vederhus et al., 2012), and the vascular reactivity may be inhibited by reduced microvascular density (Bonamy et al., 2007), and elevated circulating catecholamines (Johansson et al., 2007). Importantly, it should be noted that while attenuated vascular responses increase *T*_C_ lability and thermoeffector strain in elderly and CVD populations (Holowatz et al., 2010), it did not appear to increase cardiac stress in the childhood‐equivalent animals, as measured by duration of exposure and other cardiovascular parameters (Figure S2, Table S1).

### 4.2 Augmented SBP

The increased SBP observed is likely due to a combination of altered vascular architecture and autonomic imbalance. In healthy populations, cold exposure drives augmented sympathetic activity and vasoconstriction, yet this results in only mild central blood pressure increases, due to corresponding changes in cardiac output (Ikaheimo, 2018; Wilson & Crandall, 2011). However, in elderly populations cold exposure drives marked increases in SBP due to increased arterial stiffness and increased ventricular preload (Greaney et al., 2014; Hess et al., 2009; Wilson et al., 2010). This is a key component of the elderly cardiovascular morbidity and mortality during cold seasons (Ikaheimo, 2018). Similarly, children born preterm have been shown to possess narrowed and stiffened arteries (Edwards et al., 2014; Jiang et al., 2006; Mohlkert et al., 2017; Morsing et al., 2014; Odri Komazec et al., 2016), reduced microvascular density (Bonamy et al., 2007), excess circulating catecholamines (Johansson et al., 2007), and altered reactivity to acetylcholine (Morsing et al., 2014). Elevated SBP can reflect arterial narrowing and stiffening (Ikaheimo, 2018), both of which have been previously observed in those born preterm (Mohlkert et al., 2017; Morsing et al., 2014). Furthermore, circulating catecholamine concentration did not appear to be associated with the observed elevation of SBP in the current study (Figure S5). Importantly, as SBP influences cardiac workload and myocardial oxygen demand, this may be a driver for preterm‐associated cardiovascular risk during cold stress. On average, rate pressure product (an indirect measure of cardiac work) in preterm‐born animals was increased above that of term‐born animals, indicating greater cardiac strain for comparative decreases in *T*_C_.

Further work will be required to confirm these findings (both mechanistically and clinically), but an investigative scaffold is present in the assessment of cardiovascular responses to thermal stress in the elderly (e.g., (Hess et al., 2009; Holowatz & Kenney, 2010)). The excess SBP observed here suggests greater cardiovascular strain and therefore a greater risk of adverse events (e.g., myocardial ischemia) in response to cold exposure. Similarly, attenuated vascular responses may exacerbate heat stress, and resultant cardiovascular strain as also observed in elderly populations. It should be noted that these findings were from a young, apparently healthy (i.e., comparable resting cardiovascular and hemodynamic profile) population. If a similar profile of thermal vulnerability exists in humans born preterm, this represents another cause of latent morbidity that may become manifest as an excess of cardiovascular dysfunction as the increasing cohorts of adults born preterm approach middle and older age.

We believe that these data present an intriguing insight into the vulnerability of those born preterm to the adverse cardiovascular consequences of thermal stress. This warrants further investigation, particularly as we face a future of expanding frequency and severity of thermal extremes—both hot and cold (IPCC, 2023). Given the equivalent age and species of the present model population, future studies would benefit from investigating ex‐preterm humans across childhood, adolescence and mature adulthood. Studies would benefit from replicating the thermosensory findings of Walker, Franck, Fitzgerald, Myles, Stocks and Marlow (Walker et al., 2009), as well as examining T_C_, acuity of vasomotor reactivity, dependence of vasomotor pathways, and autonomic activity that controls blood pressure and cardiac strain.

### 4.3 Limitations

The present findings indicate a possible “latent cardiovascular risk” in those born preterm to environmental extremes, although much work will be required to confirm and explain these findings. First and foremost, this work deserves investigation in the human preterm‐born population. Utilizing our guinea pig model of preterm birth we have been able to specifically examine the effect of preterm birth per se on long‐term cardiovascular health. While a well‐developed model with good comparability to the human population (Morrison et al., 2018), it is not without its limitations. Chiefly, anesthetic widens thermoregulatory and cardiovascular thresholds. To ameliorate this effect, we minimized anesthetic dosage (Sixtus, Gray, et al., 2021), and validated the thermal stress (Sixtus, Berry, et al., 2023) to ensure thermoregulatory and cardiovascular reactivity and animal safety. Additionally, different species respond to thermal stress in different ways. However, the cardiovascular response in which we were interested remains the backbone of the thermal response among mammals, and importantly the cardiovascular response to both challenges was in line with what is known in human populations. Regarding comparisons made between the preterm‐born population and elderly individuals we do not intend to suggest the same level of vulnerability exist between populations. However, the elderly population remains the most well‐described vulnerable population in the climate change context and therefore the easiest point of comparison. Further still, it must be acknowledged that many similarities exist between the cardiovascular compromise observed in preterm‐born adults (~30 years/old) and that of the elderly population (e.g., impaired thermosensation, impaired cardiac function under stress, stiffened and narrowed arteries, microvascular rarefaction, elevated oxidative stress) (Sixtus, Gray, et al., 2023). The present results suggest that further research into the preterm risk during thermal extremes is warranted.

## 5 CONCLUSION

In conclusion, in our model of preterm birth, perturbations in the cardiovascular response to both heat and cold stress were clearly observed. These changes—attenuated dilation in response to heat, and elevated SBP in response to cold—are in line with those seen in populations already known to be vulnerable to environmental temperature fluctuation, such as the elderly. Given that pronounced cardiovascular dysfunction has been observed in adolescence and early adulthood, it is likely that the responses observed in the current study will also become more pronounced throughout life and further exacerbated by thermal extremes.

## AUTHOR CONTRIBUTIONS

*Conception and design*: Ryan Phillip Sixtus, Clint Gray, Mary Judith Berry, Rebecca Maree Dyson. *Acquisition*, *analysis or interpretation of data*: Ryan Phillip Sixtus, Emily Sarah Jane Paterson, Heather Barnes, Rebecca Maree Dyson. *Drafting and revision*: Ryan Phillip Sixtus, Clint Gray, Emily Sarah Jane Paterson, Heather Barnes, Mary Judith Berry, Rebecca Maree Dyson. *Final approval*: Ryan Phillip Sixtus, Clint Gray, Heather Barnes, Emily Sarah Jane Paterson, Mary Judith Berry, Rebecca Maree Dyson.

## FUNDING INFORMATION

RPS was supported by grants from the Heart Foundation of New Zealand, and by the Neonatal Trust (NZ). RMD was supported by a University of Otago Health Sciences Career Development Fellowship. RPS is currently supported by a BBSRC grant.

## CONFLICT OF INTEREST STATEMENT

The authors declare that they have no conflicts of interest.

## Supporting information


**Data S1:** Supporting information.

## Data Availability

The data that support the findings of this study are available from the corresponding author upon reasonable request.
