# Supplementary material for: Cardiovascular responses to heat and cold exposure are altered by preterm birth in guinea pigs
Source: Physiol Rep. 2024 Oct 22;12(20):e70098. doi: 10.14814/phy2.70098 (PMC11494451; doi:10.14814/phy2.70098)
Supplement: Supplementary file 1 — Data S1: Supporting information. [file PHY2-12-e70098-s001.docx]

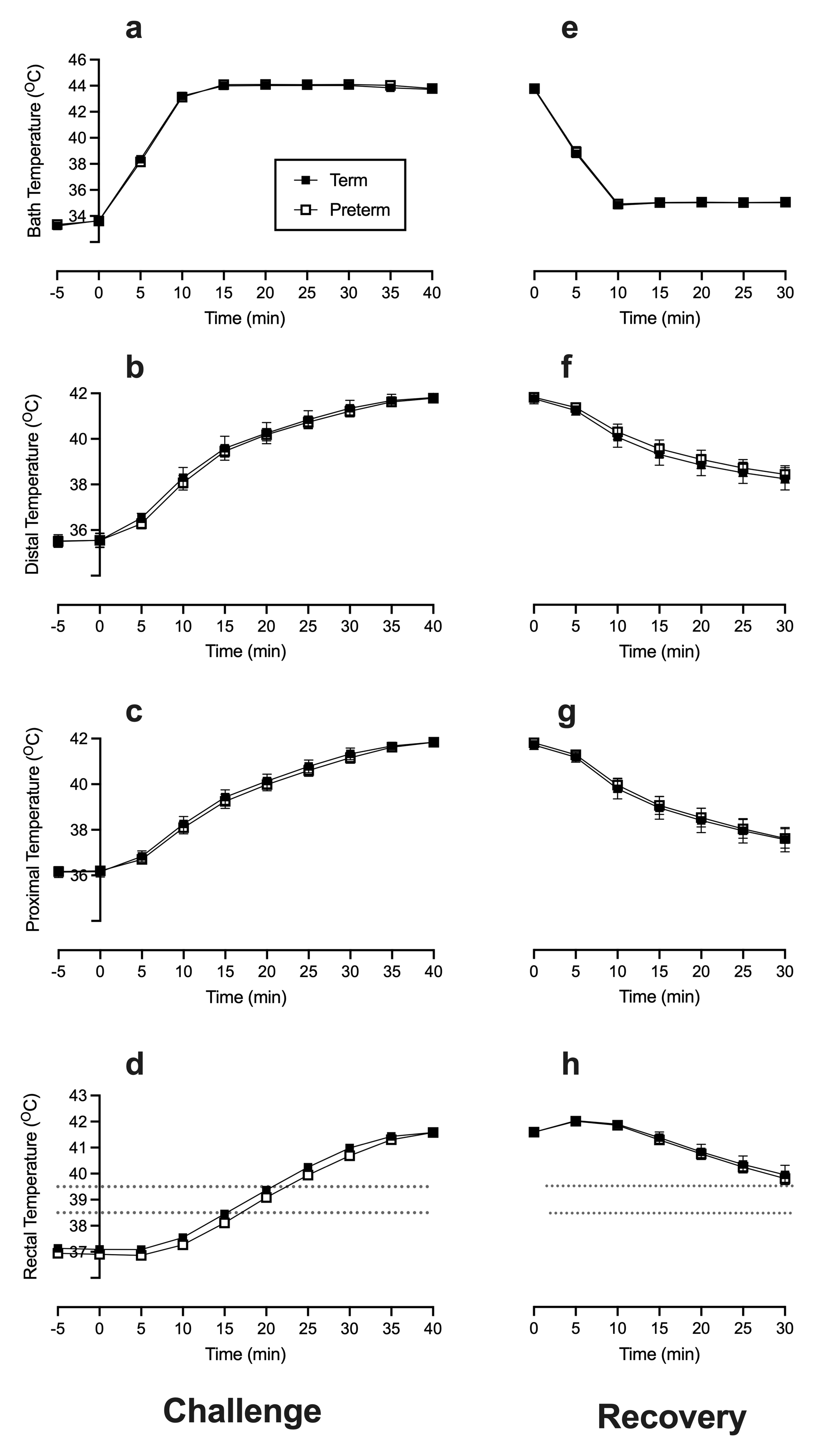


Supplemental Figure 1. Heating challenge thermal profile (mean±SD).

Panels a-d present challenge responses, and panels e-h present recovery responses. Panels a and e present the manipulated T_bath_ including the final achieved temperature. Panels b/f, and c/g present the distal and proximal skin temperature (T_sk_) response, respectively. Panels d/h present the rectal temperature (T_re_/T_c_) response; dotted lines reflect thermoneutral zone for core body temperature in guinea pigs. There was a significant effect of time, **** *P*<0.0001 on all parameters.


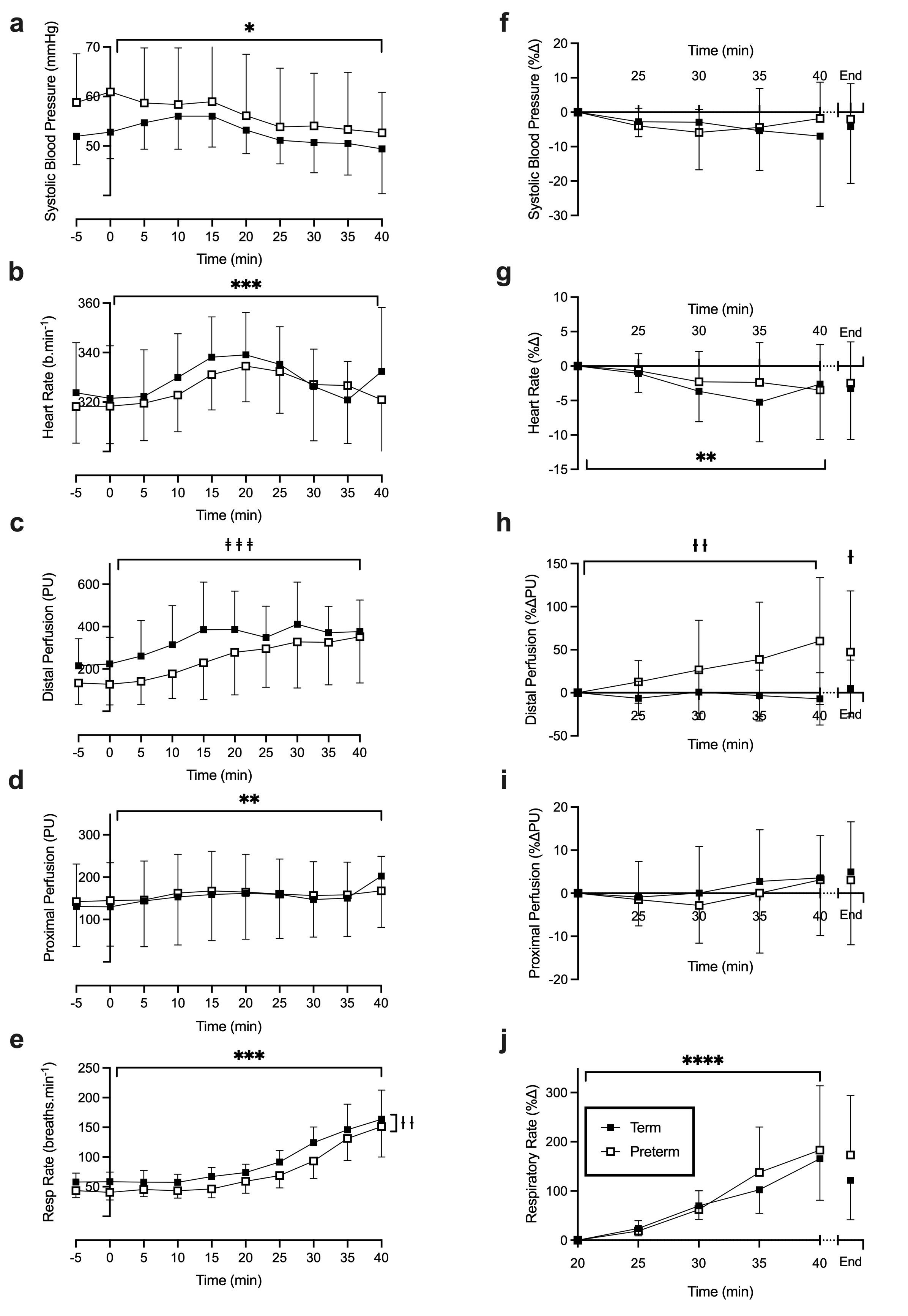


Supplemental Figure 2. Cardiovascular profile during the heating thermal challenge (mean±SD).

Panels a-e show the cardiovascular response from challenge onset, whereas panels f-j show the percent change from thermoneutral zone (T≈39^O^C) to 41.5 ^O^C. Time effect: * *P*=0.05-0.02; *** *P*=0.001-0.0002; **** *P*<0.0001. Gestation effect: $\dagger$ *P*=0.01-0.002.

Supplemental Table 1. Blood gas characteristics associated with the heating challenge.

|  |  |  | | | |  |
| --- | --- | --- | --- | --- | --- | --- |
|  |  | **Baseline** | **Post-30** | **Post-3 h** | **Post 24 hr** |  |
| Lactate | Term | 2.2 ± 1.2 | 2.9 ± 1.9 | 3.8 ± 1.8 | 2.4 ± 1.0 | * |
|  | Preterm | 2.3 ± 1.3 | 3.0 ± 1.0 | 2.9 ± 1.0 | 2.7 ± 0.9 | * |
| pH | Term | 7.47 ± 0.03 | 7.46 ± 0.04 | 7.42 ± 0.06 | 7.48 ± 0.02 | **** |
|  | Preterm | 7.45 ± 0.04 | 7.47 ± 0.05 | 7.41 ± 0.02 | 7.47 ± 0.03 | **** |
| PCO_2_ | Term | 36.0 ± 2.9 | 29.2 ± 2.9 | 34.7 ± 4.1 | 35.8 ± 2.7 | **** |
|  | Preterm | 38.4 ± 3.8 | 30.3 ± 5.6 | 36.6 ±4.9 | 38.0 ± 2.3 | ****$\dagger$ |
| PO_2_ | Term | 53.5 ± 11.7 | 61.2 ± 9.9 | 51.9 ± 5.1 | 52.0 ± 9.7 |  |
|  | Preterm | 46.1 ± 7.2 | 63.4 ± 12.1 | 47.0 ± 6.6 | 43.8 ± 7.3 |  |
| TCO_2_ | Term | 27.1 ± 2.0 | 21.4 ± 2.1 | 23.8 ± 2.8 | 27.6 ± 1.9 |  |
|  | Preterm | 27.7 ± 2.7 | 22.5 ± 2.8 | 24.3 ± 3.4 | 28.6 ± 2.2 |  |
| HCO_3_ | Term | 26.0 ± 1.9 | 20.7 ± 2.0 | 22.8 ± 2.8 | 26.5 ± 1.6 | **** |
|  | Preterm | 26.5 ± 2.6 | 21.6 ± 2.7 | 23.3 ± 3.2 | 27.5 ± 2.2 | **** |
| Base Excess | Term | 2.4 ± 2.1 | -3.3 ± 2.3 | -1.7 ± 3.6 | 2.9 ± 1.6 | **** |
|  | Preterm | 2.6 ± 2.9 | -2.2 ± 2.7 | -1.5 ± 3.4 | 3.8 ± 2.6 | **** |
| sO_2_ | Term | 88.1 ± 7.5 | 91.8 ± 3.8 | 88.6 ± 5.7 | 87.5 ± 5.9 |  |
|  | Preterm | 82.1 ± 7.3 | 91.5 ± 8.2 | 82.2 ± 6.3 | 81.9 ± 6.7 |  |
| Glucose | Term | 8.4 ± 0.8 | 9.6 ± 2.4 | 10.4 ± 2.7 | 8.8 ± 1.0 | ** |
|  | Preterm | 8.3 ± 0.8 | 9.4 ± 2.3 | 9.1 ± 1.8 | 8.7 ± 1.6 | ** |
| cTnI | Term | 0.00 ± 0.00 | -- | 0.00 ± 0.01 | 0.01 ± 0.00 |  |
|  | Preterm | 0.02 ± 0.01 | -- | 0.01 ± 0.00 | 0.00 ± 0.00 |  |
|  |  |  |  |  |  |  |

Data are presented as mean±SD. Blood gas measures were taken from arterialised capillaries immediately pre-challenge (or with blood glucose, upon fasting and pre-challenge), after 30 min recovery, and 3 hrs-, and 24 hrs post-challenge (n=16 term, n=15 preterm). Time effect: * *P*=0.05-0.02; ** *P*=0.01-0.002; **** *P*<0.0001. Gestation effect: $\dagger$ *P*=0.05-0.02. PCO_2_= pressure of arterial carbon dioxide (mmHg), PO_2_ = pressure of arterial oxygen (mmHg), TCO_2_ = total carbon dioxide (mmHg), HCO_3_ = bicarbonate (mmol/L), sO_2_ = oxygen saturation, cTnI = cardiac troponin I. Analyses performed using iStat Alinity (Abbott Point-of-Care, Princeton, USA)


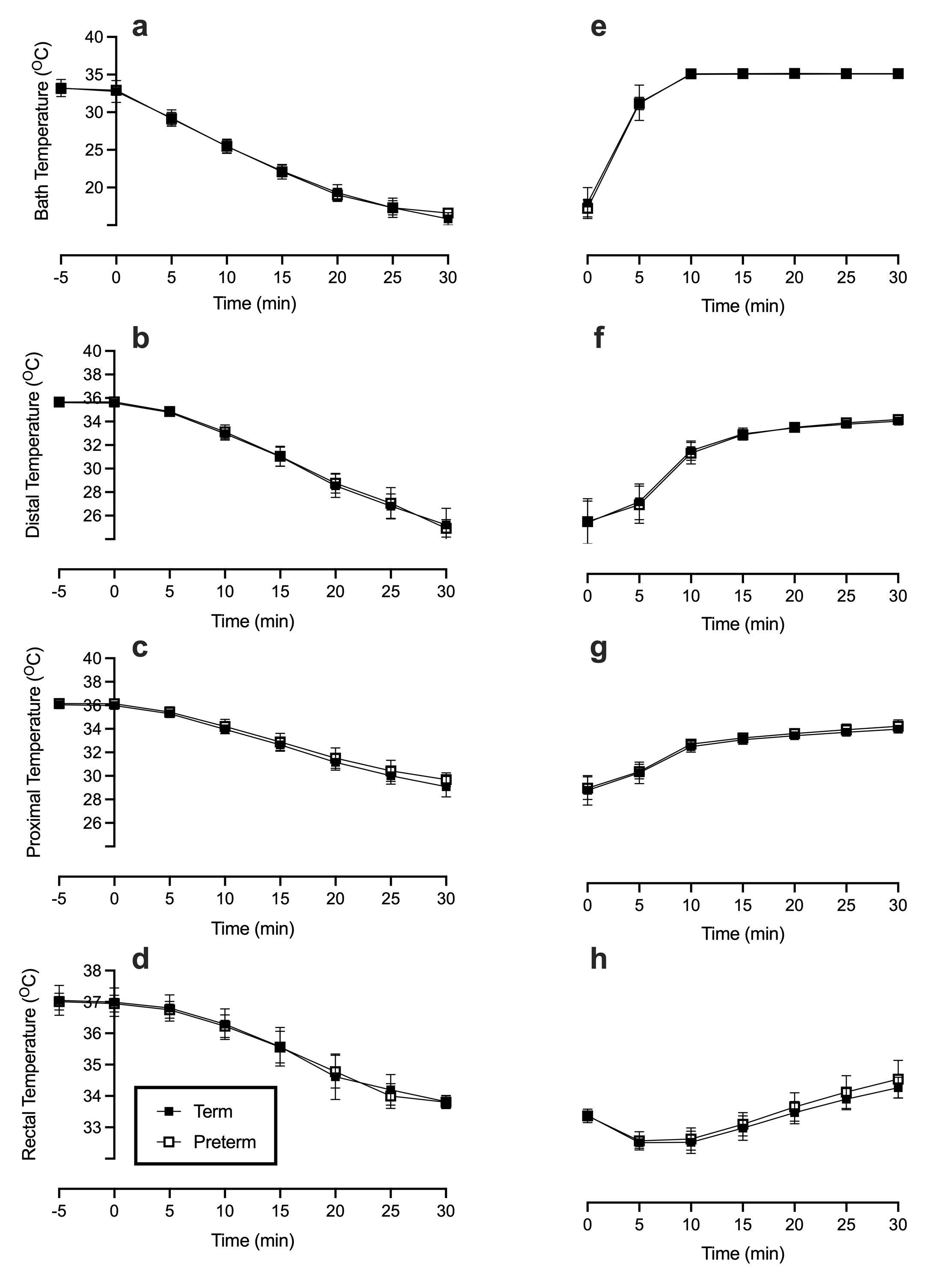


Supplemental Figure 3. Cooling challenge thermal profile (mean±SD).

Panels a-d present challenge responses, and panels e-h present recovery responses. Panels a and e present the manipulated T_bath_ including the final achieved temperature. Panels b/f, and c/g present the distal and proximal skin temperature (T_sk_) response, respectively. Panels d/h present the rectal temperature (T_re_/T_c_) response. There was a significant effect of time, **** *P*<0.0001 on all parameters.


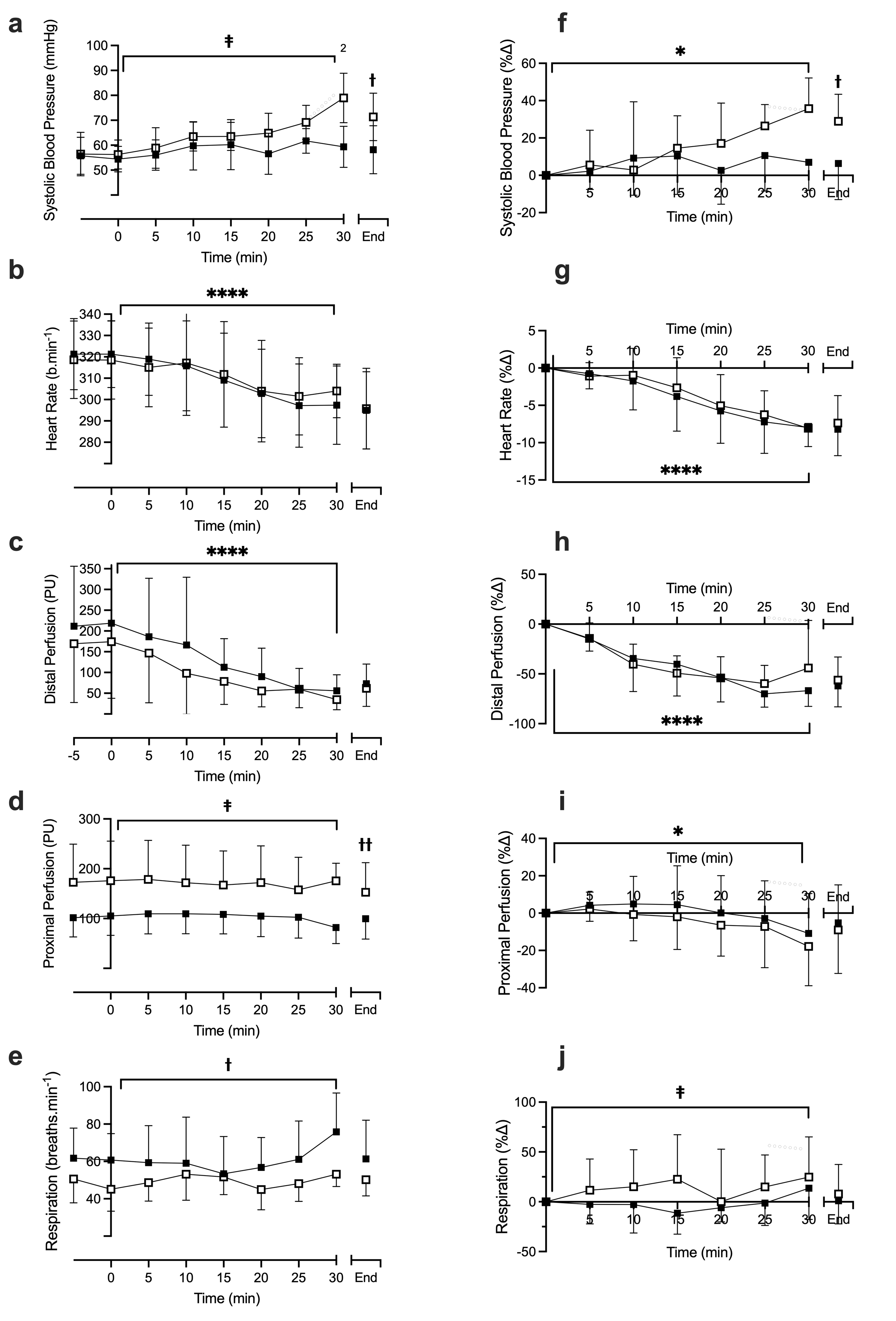


Supplemental Figure 4. Cardiovascular profile during cooling challenge (mean±SD).

Panels a-e show the cardiovascular response from baseline (-5 min) to challenge end. Panels f-j present the percentage change from challenge start to challenge end (T_re_/T_C_=34 ^O^C). Time to achieve stable hypothermic response varied, the final time of each animals’ challenge is therefore collated in the ‘end’ timepoint. Time effect: * *P*=0.05-0.02; **** *P*<0.0001. Gestation effect: $\dagger$ *P*=0.05-0.02. Interaction between gestation and time: $\ddagger$ *P*=0.05-0.02.

|  |  |  |  |  |  |  |
| --- | --- | --- | --- | --- | --- | --- |
|  |  | **Baseline** | **Post-30** | **Post-3 h** | **Post 24 hr** |  |
| Lactate | Term | 2.8 ± 1.7 | 3.6 ± 2.1 | 3.5 ± 1.9 | 3.0 ± 1.4 |  |
|  | Preterm | 2.3 ± 1.0 | 3.3 ± 1.5 | 3.2 ± 0.7 | 2.9 ± 1.1 |  |
| pH | Term | 7.47 ± 0.05 | 7.40 ± 0.04 | 7.43 ± 0.05 | 7.48 ± 0.03 | **** |
|  | Preterm | 7.46 ± 0.07 | 7.39 ± 0.04 | 7.43 ± 0.03 | 7.47 ± 0.03 | **** |
| PCO_2_ | Term | 35.1 ± 3.6 | 35.9 ± 5.3 | 34.7 ± 4.1 | 37.6 ± 2.8 | $\dagger$ |
|  | Preterm | 39.3 ± 3.6 | 39.6 ± 8.9 | 39.3 ± 7.6 | 41.1 ± 8.2 |  |
| PO_2_ | Term | 61.7 ± 26.3 | 54.8 ± 9.4 | 52.5 ± 11.3 | 47.0 ± 8.0 |  |
|  | Preterm | 40.7 ± 6.1 | 49.1 ± 11.4 | 44.8 ± 7.1 | 44.4 ± 7.3 |  |
| TCO_2_ | Term | 26.6 ± 2.9 | 23.3 ± 3.6 | 23.8 ± 2.5 | 28.9 ± 2.8 |  |
|  | Preterm | 27.8 ± 2.8 | 23.8 ± 2.9 | 25.5 ± 3.1 | 29.4 ± 2.9 |  |
| HCO_3_ | Term | 25.7 ± 3.0 | 22.3 ± 3.4 | 22.8 ± 2.4 | 27.7 ± 2.5 | **** |
|  | Preterm | 26.6 ± 2.5 | 22.5 ± 2.7 | 24.5 ± 2.9 | 28.3 ± 2.9 | **** |
| Base Excess | Term | 1.9 ± 3.5 | -2.6 ± 3.8 | -1.5 ± 2.8 | 4.2 ± 2.7 | **** |
|  | Preterm | 2.6 ± 2.8 | -2.4 ± 2.9 | 0.3 ± 3.1 | 4.6 ± 3.1 | **** |
| sO_2_ | Term | 90.4 ± 5.9 | 85.4 ± 8.6 | 86.1 ± 8.0 | 84.4 ± 6.1 |  |
|  | Preterm | 76.3 ± 8.3 | 82.4 ± 8.2 | 83.7 ± 4.9 | 84.9 ± 3.4 |  |
| Glucose | Term | 8.9 ± 1.5 | 10.3 ± 1.6 | 10.8 ± 3.5 | 9.2 ± 1.1 | * |
|  | Preterm | 8.6 ± 0.9 | 9.2 ± 1.4 | 9.5 ± 2.3 | 9.5 ± 1.8 | * |
| cTnI | Term | 0.01 ± 0.01 | -- | 0.02 ± 0.03 | 0.00 ± 0.00 |  |
|  | Preterm | 0.01 ± 0.02 | -- | 0.00 ± 0.00 | 0.00 ± 0.01 |  |
|  |  |  |  |  |  |  |

Supplemental Table 2. Blood gas characteristics associated with the cooling challenge.

Data are presented as mean±SD. All groups are n=7, except preterm males (n=6).

Blood gas measures were taken from arterialised capillaries immediately pre-challenge (or with blood glucose, upon fasting and pre-challenge), after 30 min recovery, and 3 hrs-, and 24 hrs post-challenge (n=16 term, n=15 preterm). Time effect: * *P*=0.05-0.02; **** *P*<0.0001. Gestation effect: $\dagger$ *P*=0.05-0.02. PCO_2_= pressure of arterial carbon dioxide (mmHg), PO_2_ = pressure of arterial oxygen (mmHg), TCO_2_ = total carbon dioxide (mmHg), HCO_3_ = bicarbonate (mmol/L), sO_2_ = oxygen saturation, cTnI = cardiac troponin I. Analyses performed using iStat Alinity (Abbott Point-of-Care, Princeton, USA)


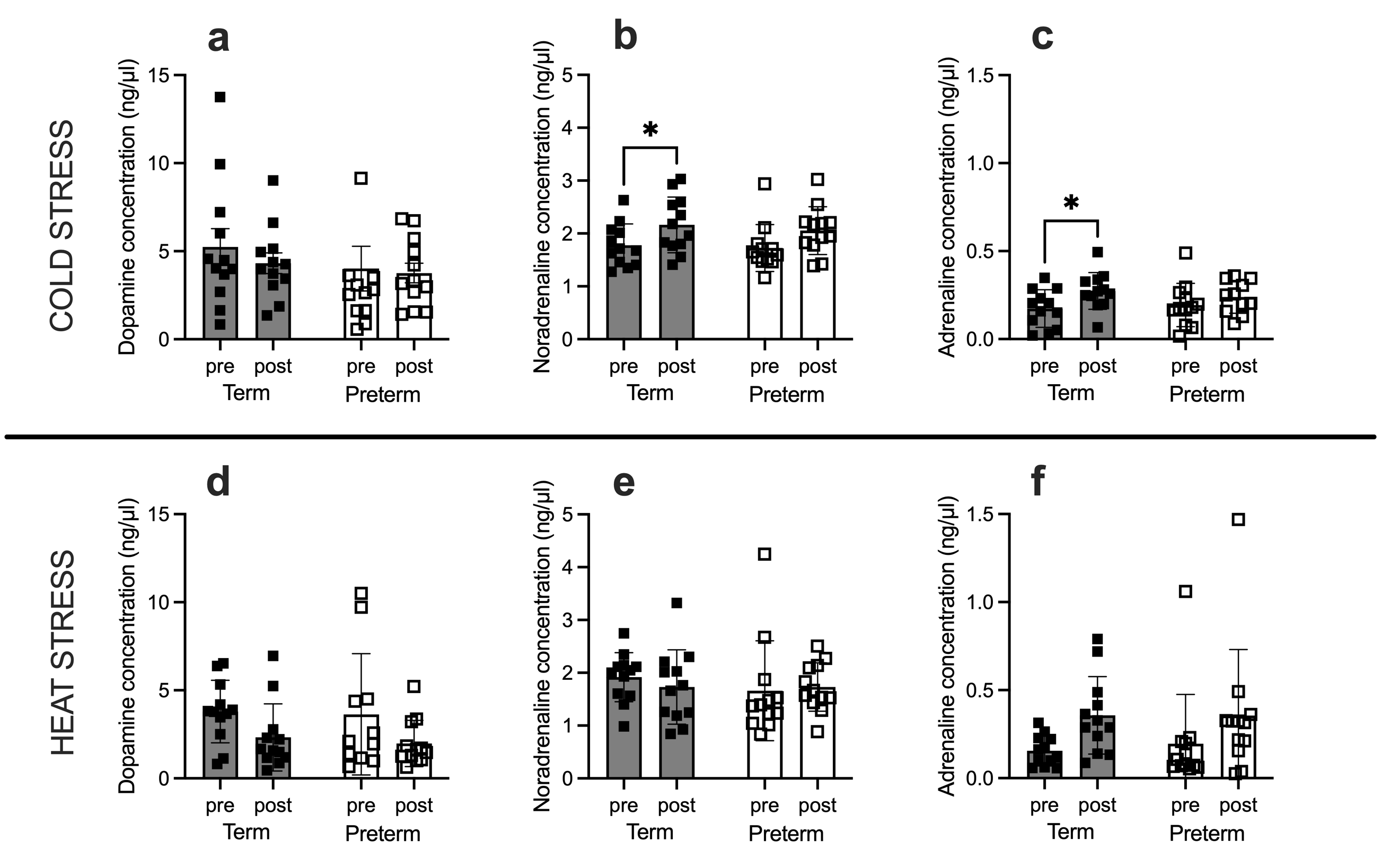


Supplemental Figure 5. Catecholamine response to heat and cold stress (mean±SD). a/d present dopamine concentrations, b/e present noradrenaline concentration, c/f present adrenaline concentration.

Time effect: * *P*=0.05-0.02. Analyses performed using 3-CAT Research ELISA (LDN, Germany)
